# Clinical outcomes of pelvic floor muscle training, electrical stimulation, and magnetic stimulation in women with postpartum stress urinary incontinence: a retrospective cohort study

**DOI:** 10.1186/s12905-026-04482-3

**Published:** 2026-05-08

**Authors:** Yanwen Qi, Cheng Sun, Lizhou Luo, Chengyuan Wang

**Affiliations:** 1 Department of Plastic Surgery, China-Japan Friendship Hospital, No. 2, Yinghua East Street, Chaoyang District, Beijing, 100029 China; 2 Department of Surgical Anesthesiology, China-Japan Friendship Hospital, No. 2, Yinghua East Street, Chaoyang District, Beijing, 100029 China

**Keywords:** Postpartum stress urinary incontinence, pelvic floor muscle training, electrical stimulation, magnetic stimulation, combined therapy, urodynamic assessment, clinical effectiveness

## Abstract

**Background:**

Postpartum stress urinary incontinence (PSUI) commonly impairs quality of life in postpartum women. Pelvic floor muscle training (PFMT), as a primary foundational treatment, faces challenges such as inadequate adherence and significant individual differences in efficacy. Electrical stimulation (ES) and magnetic stimulation (MS), as passive pelvic floor rehabilitation techniques, are often used in conjunction with PFMT in clinical settings. However, there is a lack of systematic retrospective cohort study evidence comparing the efficacy, safety, and adherence of these three methods.

**Objective:**

Compare the effects of the three intervention strategies: PFMT, PFMT combined with ES, and PFMT combined with MS, on the recovery of pelvic floor function and the improvement of clinical symptoms in patients with PSUI.

**Methods:**

This retrospective study enrolled patients with PSUI who received systematic treatment at our hospital’s plastic surgery department between June 2020 and June 2025. Patients were divided based on their treatment regimens: PF (receiving PFMT guidance), ES (receiving PFMT combined with outpatient ES therapy), and MS (receiving PFMT combined with outpatient MS therapy). A total of 102 patients (*n* = 34) were ultimately included. All patients received a 12-week course of systematic rehabilitation treatment. Primary indicators were objective measures: the amount of urine leakage during a 1-hour pad test, pelvic floor muscle strength (modified Oxford grading), and urodynamic parameters (maximum urethral closure pressure [MUCP], abdominal pressure leak point pressure [LPP]). Secondary indicators included the overall clinical response rate, International Consultation on Incontinence Questionnaire-Short Form (ICIQ-SF) scores, Incontinence Quality of Life Questionnaire (I-QOL) scores, and the incidence of adverse events.

**Results:**

Baseline characteristics were balanced among the three groups (*P* > 0.05). After 12 weeks of treatment, the urine leakage volume in all three groups decreased compared to the baseline (*P* < 0.001). Specifically, the urine leakage volume after MS and ES treatments was lower than after PF treatment (*P* < 0.01). In terms of pelvic floor muscle strength, both MS and ES treatments resulted in higher Oxford grades compared to PF (*P* < 0.001). Regarding urodynamic parameters, both MS and ES treatments showed higher MUCP and LPP values than PF (*P* < 0.001) with MS higher than ES (*P* < 0.05). In terms of clinical overall effectiveness, PF had a rate of 73.5% (25/34), ES had a rate of 91.2% (31/34), and MS had a rate of 94.1% (32/34). Both ES and MS had higher rates compared to PF (*P* < 0.05). Both MS and ES showed lower ICIQ-SF scores (*P* < 0.01) and higher I-QOL scores (*P* < 0.001) than PF. Moreover, MS had higher I-QOL scores than ES (*P* = 0.008).

**Conclusion:**

In this non-randomized study, PFMT combined with ES or MS was associated with greater improvements than PFMT alone, and MS showed more favorable urodynamic and quality-of-life changes. Causal superiority cannot be determined.

## Significance & innovation

The efficacy of a 12-week comprehensive rehabilitation program in patients with PSUI was investigated. It systematically compared the differences in efficacy between PFMT alone and the combined ES, MS, and other commonly used clinical protocols. The overall clinical effectiveness rate was used as an intuitive efficacy indicator. The findings provide preliminary comparative evidence that may inform the optimization of postpartum pelvic floor rehabilitation protocols, pending confirmation in prospective randomized studies.

## Introduction

Postpartum stress urinary incontinence (PSUI) refers to the involuntary urinary leakage that occurs in women after childbirth when there is a sudden increase in abdominal pressure. It is one of the most common types of postpartum pelvic floor dysfunction [1, 2]. Epidemiological studies indicate that the prevalence of PSUI ranges from 30% to 60%, with approximately 20% of patients experiencing varying degrees of urinary leakage symptoms even years after childbirth, significantly affecting their life [3–5]. As China’s fertility policies are adjusted and the aging population grows, the prevention and treatment of PSUI have become urgent public health issues. The primary mechanism of PSUI is related to the damage to the pelvic floor support structures [6]. The increased weight of the uterus during pregnancy and changes in hormone levels lead to the relaxation of connective tissue in the pelvic floor. During vaginal delivery, the compression of the fetal head can cause excessive stretching or even tearing of the pelvic floor muscles, fascia, and nerves, leading to a weakened function of the pelvic support structures. The position of the urethra-bladder junction also shifts downward, and the closure pressure of the urethra becomes insufficient to counteract the pressure within the bladder, resulting in urinary incontinence [7, 8]. Additionally, factors such as parity, fetal weight, mode of delivery, maternal age, and obesity are closely linked to the occurrence of PSUI [9, 10].

Currently, treatment options for PSUI primarily fall into two categories: non-surgical treatment and surgical treatment [11]. Non-surgical treatments, which serve as the primary therapeutic approach, offer advantages such as minimal invasiveness, safety, and cost-effectiveness. These treatments primarily include pelvic floor muscle training (PFMT), biofeedback, electrical stimulation (ES), magnetic stimulation (MS), and lifestyle interventions [12–14]. PFMT has been recommended as the preferred treatment option for PSUI. By instructing patients to actively contract their pelvic floor muscles, PFMT enhances muscle strength and endurance, thereby improving urinary closure function [15–17]. However, the effectiveness of PFMT heavily depends on the patient’s adherence and the correctness of the training method. Some patients experience suboptimal results due to difficulties in accurately locating their pelvic floor muscles or maintaining consistent training.

ES and MS, as passive techniques for pelvic floor rehabilitation, have been widely used in clinical practice in recent years [18, 19]. ES involves applying specific frequencies and intensities of electrical current, stimulating pelvic floor nerves and muscles, inducing passive muscle contractions, and promoting nerve function reconstruction and muscle strength recovery [20, 21]. MS utilizes time-varying magnetic fields to generate induced currents within the body, non-invasively stimulating pelvic floor nerves and muscles. It has advantages such as strong penetration, the ability to be performed without removing clothing, ease of use, and comfortable patient experience [22, 23]. Both ES and MS can improve pelvic floor muscle strength, reduce urine leakage, and enhance quality of life [24, 25]. However, there is currently a lack of systematic clinical evidence comparing the comprehensive efficacy of combining ES with MS with PFMT, particularly regarding the differences in efficacy, safety, and impact on urinary dynamics parameters between the two methods.

Therefore, the aim of this study is to conduct a retrospective cohort study to systematically compare the clinical efficacy and safety of three intervention strategies: pure PFMT, PFMT combined with ES, and PFMT combined with MS, for patients with PSUI. Emphasis will be placed on analyzing the differences between these three approaches in terms of improving pelvic floor muscle strength, reducing urine leakage, enhancing urinary dynamic parameters, improving patient quality of life, and overall clinical effectiveness. This study aims to generate hypothesis-generating evidence that may help inform the design of future prospective randomized trials and provide preliminary insights for clinical decision-making.

## Materials and methods

### Study design

**Fig. 1:**
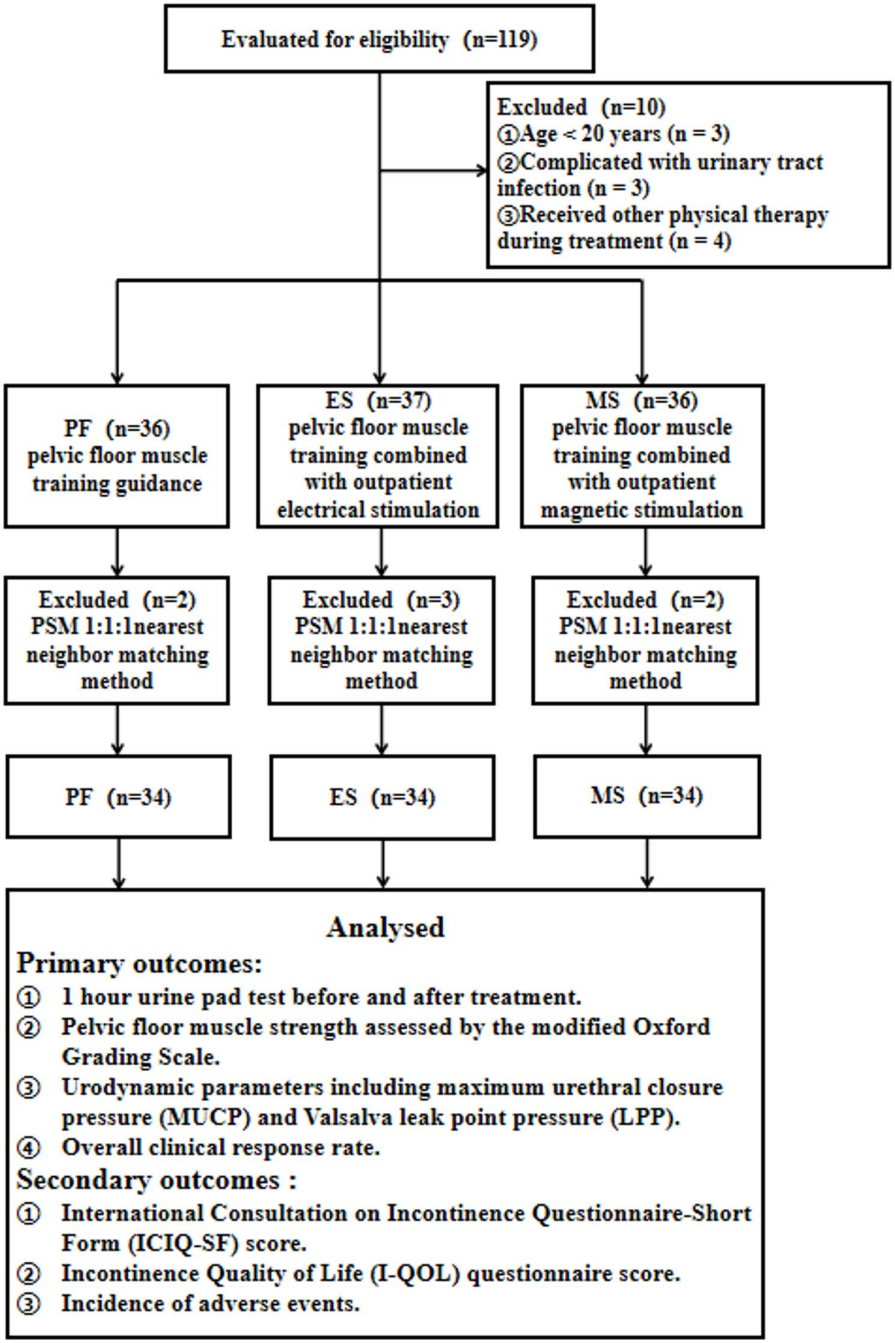
Research flowchart

This was a non-randomized retrospective comparative study. Through the hospital’s electronic medical record system, extract data on PSUI patients who received systematic treatment at our hospital’s plastic surgery department between June 2020 and June 2025. Based on the actual treatment plans received, the patients were categorized into three groups: PF (receiving PFMT guidance), ES (receiving PFMT combined with outpatient ES therapy), and MS (receiving PFMT combined with outpatient MS therapy). To reduce the confounding bias, a propensity score matching (PSM) method was employed to match the three patient groups in a 1:1:1 ratio. Ultimately, 34 patients were included in each group. The design of the workflow procedure is illustrated in Fig. 1.

### Ethical considerations

Approval was obtained from China-Japan Friendship Hospital ethics committee, and the study adhered to the Declaration of Helsinki and international ethical guidelines. Given the retrospective design using archived clinical data, the ethics committee waived the requirement for informed consent. The data were anonymized to protect participant privacy throughout the study.

### Inclusion and exclusion criteria

Inclusion criteria: ①Age 20–45 years; ②Meet the diagnostic criteria for stress urinary incontinence outlined in the *Guidelines for the Diagnosis and Treatment of Female Stress Urinary Incontinence*; ③PSUI diagnosed for the first time within 42 days to 6 months after childbirth; ④Received and completed at least 12 weeks of treatment according to the specified protocol, along with required follow-up care; ⑤Complete clinical medical records.

Exclusion criteria: ①Urinary incontinence of an urgent nature, mixed incontinence, or predominantly urgent incontinence; ②presence of urinary incontinence symptoms prior to pregnancy or a history of pelvic organ prolapse; ③co-occurrence of urinary tract infections, tumors, stones, or deformities; ④previous history of pelvic floor surgery or surgery for urinary incontinence; ⑤concurrent severe heart, lung, liver, or kidney dysfunction; ⑥concurrent administration of other physical or pharmacological treatments that may impact the evaluation of treatment efficacy during the course of treatment.

### Treatment methods

All patients received basic pelvic floor health education during their treatment, which included lifestyle guidance (avoiding heavy lifting, managing constipation, and maintaining a healthy weight), as well as instruction on proper PFMT techniques. The specific treatment plans for each group of patients are as follows:

- PF [26]: Only PFMT guidance is accepted. Trained by professional rehabilitation therapists one-on-one, patients have mastered the correct method of contracting the pelvic floor muscles (contracting the anus and vagina while avoiding the involvement of abdominal, hip, and thigh muscles). During this period, patients maintained daily training three times, each session lasting about 15 min. The training included slow contractions (contracting for 5–10 s, relaxing for 10 s, repeating 10–15 times) and fast contractions (contracting quickly for 1–2 s, relaxing for 2 s, repeating 10-15times), alternating between these methods. Training progress was monitored through phone or WeChat follow-ups every week during the 12-week treatment period.
- ES [18]: Combined ES therapy based on PF. Utilize the S480 type biofeedback electro-stimulation treatment device, equipped with vaginal electrodes. Patients are positioned in a lithotomy position, and the vaginal electrodes are inserted after disinfection. The reference electrode is placed on the abdomen. The electro-stimulation parameters are set as follows: frequency 30–50 Hz, pulse width 300–500 µs, and the current intensity is adjusted to ensure it is tolerable for the patient and can induce muscle contractions without causing pain (0–70 mA). Each session lasts 30 min, comprising 20 min of electro-stimulation and 10 min of biofeedback training. Treatments are conducted twice a week for a total of 12 weeks.
- MS [27]: Based on PF, combined MS therapy was employed. The Magneuro 100 F-type MS treatment device was used. After the patient emptied their bladder, they sat on the treatment chair with their legs naturally apart, positioning the perineum at the center of the magnetic coil on the base of the chair. All patients received standardized stimulation parameters: stimulation frequency of 30 Hz, stimulation intensity of 40%, stimulation duration of 5 s with a 5-second interval, each session lasting 20 min, with a total of 18,000 pulses. No specific discomfort was reported by the patients during the treatment. Treatments were conducted twice a week over a total of 12 weeks.
- Standardization of diagnostic and treatment pathways [28]: Diagnostic and treatment protocols were retrospectively documented as having been fully standardized. All patients underwent the same diagnostic workup (medical history, pad test, urodynamics) confirmed by two independent physicians based on the Guidelines for the Diagnosis and Treatment of Female Stress Urinary Incontinence. Within each treatment group, intervention protocols were delivered according to a prespecified manual with identical device settings, frequency, intensity, number of sessions, and follow-up schedules, as recorded in the electronic medical records. This standardization minimized within-group variability in clinical management.

### Observational parameters

Data for the following observational parameters were retrospectively extracted and collected through querying the hospital electronic medical record system, communication systems, and nursing records.

#### Baseline characteristics

Extract demographic information about the patient (age, BMI, mode of delivery, number of pregnancies, birth weight of the newborn), lifestyle habits (history of smoking, history of alcohol consumption), disease characteristics (postpartum duration, duration of urinary incontinence, severity grade of urinary incontinence), and prior treatment history (history of previous pelvic floor rehabilitation treatments, history of pelvic surgery).

#### Primary outcome measures

##### 1-hour urine pad test [29]

Urine leakage was assessed before treatment and 12 weeks after treatment. Patients emptied their bladder and then wore a pre-weighed urine pad. They drank 500 ml of water within 15 min, performed 30 min of walking, coughed 10 times, stood up and sat down 10 times, bent over to pick up an object 5 times, jogged in place for 1 min, and washed their hands with running water for 1 min. The urine pad was weighed after 1 h and the results were calculated.

##### Pelvic floor muscle strength (modified Oxford grading system) [30] 

The assessment was performed by the same experienced rehabilitation therapist. The patient was placed in the lithotomy position. The therapist placed their index and middle fingers inside the vagina and instructed the patient to contract the pelvic floor muscles. The contraction strength was graded from 0 to 5, with grades ≥ 3 defined as normal muscle strength. Due to the retrospective design, the assessor was not blinded to group allocation, which may introduce bias.

##### Urodynamic parameters [31]

The urinary dynamic analyzer Nidoc 970 A + was used for pre- and post-treatment assessments. The patient was positioned in lithotomy position. A double-lumen pressure-measuring catheter was inserted through the urethra, and a pressure-measuring catheter for abdominal pressure was inserted through the rectum. The bladder was perfused at a constant rate of 50 ml/min to record the maximum urethral closure pressure (MUCP) and the abdominal pressure leak point pressure (LPP).

##### Overall clinical efficacy rate [14]

Evaluated after 12 weeks of treatment by a trained physician who was not involved in the patients’ treatment decisions. Due to the retrospective design, the assessor was not blinded to group allocation. The definitions were as follows: Markedly effective – complete disappearance of urinary incontinence symptoms with substantial gain in quality of life; effective – significant symptom improvement with occasional incontinence not affecting daily life; ineffective – no improvement or worsening of symptoms. Overall effectiveness rate = (Markedly effective + Effective) / Total × 100%. This composite outcome was included because it reflects clinically meaningful improvement as perceived by both patients and clinicians, complementing the objective measures. This composite outcome was included because it reflects clinically meaningful improvement as perceived by both patients and clinicians, complementing the objective measures.

#### Secondary outcome measures

##### International Consultation on Incontinence Questionnaire – Short Form (ICIQ-SF) score [29]

To be completed by the patient before and after treatment. It includes three dimensions: frequency of incontinence, amount of incontinence, and impact on daily life. The total score ranges from 0 to 21 points. A higher score indicates a direct link to greater symptom severity.

##### Incontinence Quality of Life Questionnaire (I-QOL) score [4]

Completed by patients before and after treatment. It includes three dimensions: behavioral restrictions, psychosocial impact, and social embarrassment. It employs a Likert 5-point rating scale, with total scores ranging from 0 to 100. Higher scores reflect better quality of life.

##### Safety indicators [6]

Record all adverse reactions that occur during the course of treatment, such as vaginal discomfort/pain, abdominal discomfort, skin allergies/irritations, and muscle soreness.

### Statistical analysis

The data were analyzed using SPSS 26.0 software. To reduce confounding bias, we performed 1:1:1 PSM among the three treatment groups. The propensity score model included the following baseline covariates: age, body mass index (BMI), delivery mode, parity, neonatal birth weight, postpartum time at enrollment, and baseline 1-hour pad test weight [32–34]. A logistic regression model was used to estimate propensity scores. Patients were then matched using a nearest-neighbor matching algorithm with a caliper width of 0.2 times the standard deviation of the logit-transformed propensity score. Matching was performed without replacement. After matching, standardized mean differences (SMDs) were calculated to assess balance across the three groups; an SMD < 0.1 was considered indicative of good balance. Due to the retrospective nature, data completeness was verified. No missing data were present for the primary and secondary outcomes in the final matched sample. Therefore, no imputation methods were applied. Normality of the data was assessed using the Shapiro-Wilk test, and homogeneity of variance was assessed using the Levene test. Data that met the criteria of normal distribution and homogeneity of variance were represented as mean ± standard deviation. To evaluate the effects of treatment over time and between groups, a two-way repeated measures analysis of variance (ANOVA) was performed with group (PF, ES, MS) as the between-subject factor and time (baseline, 12 weeks) as the within-subject factor for each continuous outcome (1-hour pad test, Oxford grade, MUCP, LPP, ICIQ-SF, I-QOL). Post-hoc pairwise comparisons were conducted using Bonferroni correction. To control for type I error due to multiple primary outcomes (four: pad test, Oxford grade, MUCP, LPP), the corrected significance threshold was set at α = 0.0125 (0.05/4). Secondary outcomes (ICIQ-SF, I-QOL, clinical efficacy rate) are reported without adjustment and are considered exploratory. Count data were represented as counts (percentages), and comparisons between groups were conducted using chi-square tests or Fisher’s exact tests. When the chi-square test indicated a statistically significant overall difference, further pairwise comparisons were performed using the Bonferroni method to adjust the significance level. All statistical tests were two-sided, and *P* < 0.05 was considered to indicate statistical significance. Given the non-randomized, retrospective design, the comparisons presented herein are exploratory and unadjusted for potential confounders beyond those balanced by propensity score matching. Therefore, results should be interpreted as hypothesis-generating rather than confirmatory. Causal inferences cannot be drawn from these analyses.

## Results

### Baseline data comparison

As shown in Table 1, the three groups were balanced and comparable in terms of demographic characteristics such as age, BMI, occupation status, etc. Regarding obstetric characteristics, no significant between-group differences were observed in delivery mode, parity, birth weight of the newborn, rate of episiotomy/perineal laceration, use of labor analgesia, and breastfeeding rates. In terms of clinical characteristics, the distribution of postpartum time and the time of first detection of urinary incontinence were evenly distributed. With regard to lifestyle and comorbidities, no significant between-group differences were observed in smoking history, drinking history, history of chronic constipation, history of chronic cough, habitual heavy physical labor, and regular exercise habits (all *P* > 0.05), and all SMD were below 0.1, indicating good comparability.

**Table 1:** Baseline Demographic and Clinical Characteristics

Characteristic	PF (*n* = 34)	ES (*n* = 34)	MS (*n* = 34)	F/χ²	*P*
Demographic Characteristics					
Age (x̄±s, years)	29.85 ± 3.56	30.21 ± 3.94	29.59 ± 3.47	0.246	0.783
BMI (x̄±s, kg/m^2^)	22.91 ± 2.39	23.26 ± 2.66	22.74 ± 2.40	0.387	0.680
Employment status (*n*, %)				0.258	0.879
-Employed	22 (64.71)	23 (67.65)	21 (61.76)		
-Homemaker	12 (35.29)	11 (32.35)	13 (38.24)		
Obstetric Characteristics					
Delivery mode (*n*, %)				0.283	0.868
-Vaginal delivery	24 (70.59)	23 (67.65)	25 (73.53)		
-Cesarean section	10 (29.41)	11 (32.35)	9 (26.47)		
Parity (*n*, %)				0.243	0.886
-Primiparous	20 (58.82)	19 (55.88)	21 (61.76)		
-Multiparous	14 (41.18)	15 (44.12)	13 (38.24)		
Neonatal birth weight (x̄*±s*, g)	3320.50 ± 380.23	3403.26 ± 398.11	3350.47 ± 390.58	0.393	0.676
Episiotomy/perineal laceration (*n*, %)	15 (44.12)	16 (47.06)	14 (41.18)	0.239	0.888
Labor analgesia use (*n*, %)	18 (52.94)	17 (50.00)	19 (55.88)	0.236	0.889
Breastfeeding (*n*, %)	28 (82.35)	27 (79.41)	29 (85.29)	0.405	0.817
Clinical Characteristics					
Postpartum time (x̄*±s*, weeks)	10.56 ± 4.21	10.24 ± 4.05	10.79 ± 4.52	0.143	0.867
Time of first incontinence detection (n, %)				0.609	0.738
-Within 42 days postpartum	22 (64.71)	21 (61.76)	24 (70.59)		
-42 days to 3 months postpartum	12 (35.29)	13 (38.24)	10 (29.41)		
Lifestyle and Comorbidities					
Smoking history (*n*, %)	2 (5.88)	1 (2.94)	2 (5.88)	0.053	0.974
Alcohol consumption history (*n*, %)	3 (8.82)	2 (5.88)	4 (11.76)	0.274	0.872
Chronic constipation history (*n*, %)	5 (14.71)	6 (17.65)	4 (11.76)	0.469	0.791
Chronic cough history (*n*, %)	4 (11.76)	5 (14.71)	5 (14.71)	0.021	0.990
Heavy physical labor habit (*n*, %)	6 (17.65)	5 (14.71)	6 (17.65)	0.141	0.932
Regular exercise habit (*n*, %)	10 (29.41)	11 (32.35)	9 (26.47)	0.283	0.868

*BMI* Body Mass Index

### Urine pad test

As shown in Table 2, after 12 weeks of treatment, the urinary leakage volume for both ES and MS was lower than that for PF (*P* < 0.01). The urinary leakage volume for MS decreased to (1.71 ± 0.68) g, outperforming ES (2.12 ± 0.77) g. When examining improvements within each group, there were highly significant differences before and after treatment in all three groups (*P* < 0.001). This suggests that combined physical therapy may be associated with better control of urine flow, although causal inference cannot be made.

**Table 2:** Comparison of 1-Hour Pad Test Outcomes (g, x̄±s)

Group	Baseline	12 weeks
PF (*n* = 34)	8.56 ± 2.18	2.85 ± 1.02
ES (*n* = 34)	8.29 ± 2.07	2.12 ± 0.77^a^
MS (*n* = 34)	8.79 ± 2.42	1.71 ± 0.68^b^

Repeated measures ANOVA group × time F = 8.44, *P* < 0.001

^a^ *P* < 0.01 vs. PF

^b^ *P* < 0.001 vs. PF; MS vs. ES: *P* = 0.045 (ns after Bonferroni)

### Pelvic floor muscle strength

As shown in Table 3, intergroup comparisons after treatment revealed that the pelvic floor muscle strength of both ES and MS was higher (*P* < 0.001). This suggests that combining ES or MS with PFMT may be associated with greater improvement in pelvic floor muscle strength, although the effects of the two physical treatment methods are comparable in terms of enhancing muscle strength. Clinically, the mean muscle strength after PF treatment (grade 2.35) remained below the normal muscle strength threshold (grade 3), whereas ES (grade 3.09) and MS (grade 3.21) had already reached normal levels. This indicates that while individual home training may be effective to some extent, it is unlikely to enable most patients to regain normal muscle strength. Conversely, combining physical therapy can help patients cross the critical threshold of “normal muscle strength” and lay the foundation for improved urinary control abilities.

**Table 3:** Comparison of Pelvic Floor Muscle Strength (grade, x̄±s)

Group	Baseline	12 weeks
PF (*n* = 34)	1.53 ± 0.56	2.35 ± 0.66
ES (*n* = 34)	1.62 ± 0.60	3.09 ± 0.62^a^
MS (*n* = 34)	1.59 ± 0.61	3.21 ± 0.69^b^

Repeated measures ANOVA: group × time F = 14.60, *P* < 0.001

^a^ *P* < 0.01 vs. PF

^b^ *P* < 0.001 vs. PF, MS vs. ES, *P* = 0.48 (ns after Bonferroni)

### Urodynamic parameters

As shown in Table 4, comparisons between the treatment groups indicate that both ES and MS exhibited higher MUCP values compared to PF (*P* < 0.001), with MS showing a higher value than ES (*P* = 0.010). This result suggests that the combined effects of physical therapy may be associated with greater improvement in intrinsic sphincter function compared with home-based training alone, and MS showed more favorable changes. Both ES and MS exhibited higher LPP values compared to PF (*P* < 0.001), and MS showed a higher value than ES (*P* = 0.006). ES and MS reached a satisfactory level of resistance to pressure. Overall, both ES and MS were associated with improved urethral closure function and resistance capability, with MS showing more favorable changes. One speculative hypothesis is that these differences might be related to the non-invasive and deep penetration characteristics of MS, but this mechanism was not directly measured in the present study and requires further investigation. Clinically, a MUCP above 60 cmH₂O and an LPP above 100 cmH₂O are considered thresholds for adequate urethral closure function. In this study, only the ES and MS groups exceeded both thresholds, with MS achieving the highest values (MUCP 74.82 ± 8.01 cmH₂O; LPP 119.74 ± 13.05 cmH₂O), indicating clinically meaningful improvement.

**Table 4:** Comparison of Urodynamic Parameters (x̄±s)

Group	Baseline	12 weeks
MUCP (cmH_2_O)		
PF (*n* = 34)	36.38 ± 4.59	53.74 ± 6.21
ES (*n* = 34)	35.74 ± 4.24	69.47 ± 7.78^a^
MS (*n* = 34)	36.03 ± 4.81	74.82 ± 8.01^b^
LPP (cmH_2_O)		
PF (*n* = 34)	80.32 ± 8.18	95.21 ± 10.79
ES (*n* = 34)	79.85 ± 8.21	110.29 ± 12.88^a^
MS (*n* = 34)	81.06 ± 8.43	119.74 ± 13.05^b^

*MUCP* Maximum Urethral Closure Pressure, *LPP* Leak Point Pressure, MUCP, ^a^ *P* < 0.01 vs. PF, ^b^ *P* < 0.001 vs. PF, MS vs. ES *P* = 0.010 (significant after Bonferroni).* LPP*, ^a^ *P* < 0.01 vs. *PF* ^b^ *P* < 0.001 vs. *PF*, MS vs. ES *P* = 0.006

### Subjective symptoms and quality of life

As shown in Table 5, all three groups improved significantly from pre-treatment (*P* < 0.001), indicating that all three intervention strategies effectively alleviated patients’ subjective symptoms and improved their quality of life. In terms of the ICIQ-SF scores, the inter-group comparison after treatment revealed that both ES and MS scores were lower than PF (*P* < 0.01). Regarding the I-QOL scores, the inter-group comparison showed that both ES and MS scores exceeded PF (*P* < 0.001) and that MS scores were higher than ES (*P* = 0.008). Overall, both electrical and MS were associated with improved symptoms and quality of life, with MS showing more favorable changes in quality of life scores. This may be partly related to the non-invasive and comfortable treatment experience of MS, although this remains speculative.

**Table 5:** Comparison of ICIQ-SF and I-QOL Scores

Group	Baseline	12 weeks
ICIQ-SF		
PF (*n* = 34)	13.29 ± 2.86	5.76 ± 2.13
ES (*n* = 34)	12.97 ± 2.59	4.26 ± 1.71^a^
MS (*n* = 34)	13.41 ± 2.64	3.68 ± 1.59^b^
I-QOL		
PF (*n* = 34)	58.62 ± 8.28	72.41 ± 9.35
ES (*n* = 34)	60.32 ± 9.03	82.12 ± 10.01^a^
MS (*n* = 34)	58.47 ± 8.69	89.56 ± 10.57^b^

*ICIQ-SF* International Consultation on Incontinence Questionnaire-Short Form, *I-QOL* Incontinence Quality of Life Questionnaire, *ICIQ-SF *^a^ *P* < 0.01 vs. PF, ^b^ *P* < 0.001 vs. PF, ES vs. MS, *P* = 0.393 (ns). *I-QOL* ^a^ *P* < 0.01 vs. PF, ^b^ *P* < 0.001 vs. PF, MS vs. ES *P* = 0.008

### Clinical efficacy

As shown in Table 6, the overall clinical efficacy rates of ES and MS were higher than those of PF (91.18% vs. 73.53%, *P* < 0.05; 94.12% vs. 73.53%, *P* < 0.01). Analysis of treatment outcomes revealed that the proportion of patients meeting the “markedly effective” criteria for ES and MS was higher than for PF (52.94% vs. 29.41%, *P* < 0.05 ; 58.82% vs. 29.41%, *P* < 0.01), while the proportion of patients who did not respond to treatment was lower than for PF (8.82% vs. 26.47%, *P* < 0.05, 5.88% vs. 26.47%, *P* < 0.01 ). This finding not only confirms the superiority of combined physical therapy in terms of overall efficacy but also highlights its advantages in terms of the depth of treatment outcomes: combined therapy not only increases the rate of effective responses but, more importantly, enhances the quality of treatment, enabling more patients to achieve the markedly effective response level. This is of great significance for improving patients’ long-term prognosis.

**Table 6:** Comparison of Clinical Efficacy

Group	Markedly Effective	Effective	Ineffective	Total Effective Rate
PF (*n* = 34)	10 (29.41)	15 (44.12)	9 (26.47)	25 (73.53)
ES (*n* = 34)	18 (52.94)	13 (38.24)	3 (8.82)	31 (91.18)
MS (*n* = 34)	20 (58.82)	12 (35.29)	2 (5.88)	32 (94.12)
*χ²*				7.120
*P*				0.028

### Safety analysis

As shown in Table 7, all adverse events were mild and could resolve on their own or with symptomatic treatment, without any severe adverse events necessitating discontinuation of treatment. No significant differences were found in adverse events among the three groups (*P* > 0.05). However, the overall number of adverse events was very low, which limits the statistical power to detect differences in safety profiles. Therefore, we cannot conclude that the three interventions are equivalent in terms of safety. All three protocols appeared to be generally well tolerated in this small sample, but larger studies are needed to confirm their comparative safety.

**Table 7:** Comparison of Adverse Events

Adverse Reaction	PF (*n* = 34)	ES (*n* = 34)	MS (*n* = 34)	χ²	*P*
Vaginal discomfort/pain	1 (2.94)	4 (11.76)	1 (2.94)	1.461	0.482
Abdominal discomfort	0 (0.00)	1 (2.94)	1 (2.94)	0.128	0.938
Skin allergy/irritation	1 (2.94)	2 (5.88)	0 (0.00)	0.773	0.680
Muscle soreness	2 (5.88)	1 (2.94)	1 (2.94)	0.065	0.968

## Discussion

This study systematically compared the clinical efficacy and safety of the PF, ES, and MS approaches for patients with PSUI. All three approaches were effective in improving patients’ urinary incontinence symptoms, pelvic floor muscle strength, urodynamic parameters, and quality of life. However, in this study, the combined use of physical therapy was associated with greater improvements compared with home-based training alone, and the MS group **showed more favorable changes in several indicators. Due to the non-randomized design, these findings should be interpreted as associations rather than causal effects.

The study found that ES and MS outperformed PF in terms of urinary incontinence volume, pelvic floor muscle strength, urodynamic parameters, and quality of life after treatment. The overall effectiveness rates were 91.18% and 94.12%, respectively, compared to PF (73.53%). This outcome aligns with previous research trends [35, 36]. While home-based PFMT is a recommended first-line approach, its effectiveness depends to some extent on the patient’s adherence and training skills. Some patients may experience reduced efficacy due to difficulties in accurately locating the pelvic floor muscles or maintaining consistent training. ES and MS, as passive rehabilitation techniques, directly activate pelvic floor neuromuscular units through external energy input, potentially compensating for the limitations of active training [37, 38]. In the present study, patients with ES and MS did not need to rely solely on their own muscle contraction techniques to achieve muscle activation. This could be a potential mechanism, but the influence of differential supervision cannot be ruled out.

In terms of pelvic floor muscle strength, ES and MS yielded comparable results, with post-treatment averages of 3.09 and 3.21, respectively, both exceeding the normal muscle strength threshold (≥ 3). In contrast, PF treatment resulted in an average of 2.35, still below normal levels. Regarding urodynamic parameters, MS showed superior improvements in MUCP and LPP compared to ES. In terms of subjective quality of life, MS had a higher I-QOL score than ES, approaching the level of normal individuals. One speculative explanation for these differences is that the two physical therapies may have distinct mechanisms of action. ES involves the generation of an electric current through the insertion of electrodes into the vagina, directly stimulating the pelvic floor nerves and muscles, but the depth of penetration is relatively limited, and vaginal insertion may cause discomfort in some patients [39, 40]. MS utilizes time-varying magnetic fields to generate induced currents within the body, requiring no electrode insertion, and has a stronger penetrating ability, allowing for non-invasive stimulation of deep pelvic tissues [41]. The performance of MS in terms of urodynamics and quality of life in this study might be associated with its non-invasive nature and patient acceptance, but this remains speculative as these mechanisms were not directly measured. Genetic studies have identified specific collagen gene polymorphisms (e.g., COL3A1 rs1800255) associated with SUI, suggesting that individual connective tissue susceptibility may influence the pathophysiology of PSUI and potentially affect treatment response [42]. Additionally, patients with PSUI often experience varying degrees of psychological stress. Research indicates that the cortisol signaling pathway plays a crucial role in the pathophysiological processes induced by stress, potentially influencing tissue repair and inflammatory responses and thus contributing to the development of various diseases [43]. In this study, the combined use of physical therapy was associated with greater improvement in quality of life for individuals in the MS group. Beyond objective benefits, the potential link between a more comfortable treatment experience and reduced psychological stress warrants further investigation.

In terms of efficacy grading, the proportion of MS and ES meeting the “markedly effective” criteria was higher than that of PF. This suggests that combined physical therapy not only enhances the rate of effective responses but may also enable more patients to experience significant symptom relief. From the perspective of functional recovery, the mean muscle strength after PF treatment remained below the normal muscle strength threshold, while ES and MS had already reached normal levels. In terms of urinary dynamics, the LPP after PF treatment was slightly higher than the generally accepted normal lower limit, while ES and MS had achieved a good level of resistance to pressure. These results indicate that while solo home training may be effective to some extent, combined physical therapy was associated with a higher proportion of patients reaching functional recovery in this study [27]. The incidence rates of adverse events were all relatively low and mild for all three groups, with no instances of severe adverse events necessitating discontinuation of treatment. The incidence of vaginal discomfort/pain was slightly higher for ES compared to PF and MS; however, this difference was not statistically significant. The incidence of adverse events in MS was similar to that in PF, and no additional safety risks were observed.

There are limitations to this study: First, the follow-up period was only 12 weeks, resulting in a lack of long-term efficacy data. Therefore, data on the durability of treatment effects and the risk of recurrence are unavailable. This limits our ability to assess long-term outcomes. Second, the PFMT-alone group received home-based, unsupervised training, whereas the ES and MS groups received supervised, clinic-based therapy. This difference in treatment setting and supervision level, rather than the specific modalities themselves, may have contributed to the observed outcome differences. Moreover, no quantitative assessment of adherence was available for any group, precluding adjustment for this bias. Third, the sample size (34 patients per group) is modest, which may limit the power of subgroup analyses and prevent a thorough exploration of individual differences in efficacy. Fourth, no multivariable adjustment was performed beyond PSM; thus, residual confounding cannot be excluded. All findings are exploratory and require confirmation in prospective studies. Additionally, selection bias may have been introduced because treatment allocation was based on patient preference and clinical availability rather than randomization, despite propensity score matching. Fifth, the clinical efficacy rate is a subjective composite outcome and was assessed without blinding to group allocation, which may introduce bias. Similarly, the assessment of pelvic floor muscle strength using the modified Oxford grading scale was also performed without blinding, potentially introducing assessor bias. Therefore, we emphasize the objective outcomes (pad test, MUCP, LPP) as the primary findings. Sixth, this study applied strict inclusion criteria (e.g., exclusion of patients with comorbidities, prior pelvic floor surgery, mixed incontinence, or age > 45 years). Therefore, the results apply only to young, relatively healthy postpartum women with pure stress urinary incontinence and may not be generalizable to broader clinical populations. Seventh, the number of adverse events was very low across all groups, which limits statistical power for safety comparisons. Therefore, no conclusion of safety equivalence among the three interventions can be drawn. Future research with a prospective, large-sample, and long-term follow-up design will be necessary to further validate the conclusions of this study.

## Conclusion

In summary, in this non-randomized retrospective study, combining ES or MS with PFMT was associated with improved clinical outcomes for PSUI compared with PFMT alone. The MS group was associated with more favorable changes in urodynamic parameters and quality of life. These associations suggest potential benefits, but causal superiority cannot be concluded from this study design. Prospective randomized trials are needed to confirm these findings.

## Data Availability

The data supporting the findings of this study can be obtained from the corresponding author, upon request.

## Abbreviations

ANOVA: Analysis of variance
BMI: Body mass index
ES: Electrical stimulation
ICIQ-SF: International Consultation on Incontinence Questionnaire-Short Form
I-QOL: Incontinence Quality of Life Questionnaire
LPP: Leak point pressure (abdominal pressure leak point pressure)
MS: Magnetic stimulation
MUCP: Maximum urethral closure pressure
PF: Pelvic floor muscle training alone (the PF group)
PFMT: Pelvic floor muscle training
PSM: Propensity score matching
PSUI: Postpartum stress urinary incontinence
SMD: Standardized mean difference
